# Amoebic liver abscess: an unusual cause for a right iliac fossa mass: a case report

**DOI:** 10.1186/s12879-016-2093-y

**Published:** 2016-12-08

**Authors:** Vithiya Ratnasamy, Kumanan Thirunavukarasu, Kannathasan Selvam, Murugananthan Arumugam

**Affiliations:** 1 University Medical Unit, Teaching Hospital Jaffna, Jaffna, Sri Lanka; 2 Division of Parasitology, Department of Pathology, Faculty of Medicine, University of Jaffna, Jaffna, Sri Lanka

**Keywords:** Amoebiasis, Liver abscess, Right iliac fossa mass, Peritonitis, Case report

## Abstract

**Background:**

Amoebic liver abscess is the most common extra intestinal manifestation of amoebiasis in tropical countries. It usually presents with right hypochondrial pain, fever and anorexia. Amoebic liver abscess has gained clinical significance due to the wide variety of clinical presentations which can cause diagnostic dilemmas and high mortality in untreated cases.

**Case presentation:**

We report a case of a 63-year-old male with a history of anorexia for 3 weeks, fever for 4 days and examination findings of tender hepatomegaly with a liver span of 15 cm in the mid clavicular line and a firm irregular mass in the right iliac fossa. Ultrasound scan of the abdomen showed two large liver abscesses with one of them leaking into the peritoneal cavity causing a localized pus collection, which had been walled off in the right iliac fossa. He was treated with metronidazole and liver abscesses were drained percutaneously under ultrasound scan guidance. The diagnosis of *Entamoeba histolytica* infection was confirmed with the serology and subsequently by PCR from the aspirated material. He made an uneventful recovery with resolution of the symptoms and right iliac fossa mass.

**Conclusion:**

Recognition of variable presentation of amoebic liver abscess is vital, considering the curable nature of this disease and potentially fatal outcome of untreated abscess. An intra-abdominal mass in a patient with amoebic liver abscess should raise the suspicion of a localized collection of pus and impending generalized peritonitis. Early diagnosis and prompt intervention can prevent the dreaded complication of peritonitis and toxemia, and hence reduce the consequent morbidity and mortality.

## Background

Amoebiasis is a common endemic condition in South East Asian countries. It results from the infection with the protozoan parasite *Entamoeba histolytica*. Amoebic Liver Abscess (ALA) is the most common extra intestinal entity of invasive amoebiasis [1]. It is estimated that the above parasitic condition is responsible for an annual death rate of 100,000 people globally and the fatality is mainly due to the complications of amoebic colitis and amoebic liver abscess [2]. The protozoa have gained clinical significance worldwide in the recent era due to increased travel into and from tropical countries, and variable presentations of the disease which causes diagnostic confusion in the non endemic areas. ALA commonly presents as an acute illness with right hypochondrial pain, fever and anorexia [3]. *Entamoeba histolytica* infection is transmitted by ingestion of fecally contaminated food or water. Malnutrition, poor sanitation, alcoholism, immunosuppression, recent travel to endemic area, and poor socio- economic status have been identified as potential risk factors [4]. Studies have reported that the ultrasound scan has a sensitivity of more than 90% for detecting ALA and it is highly recommended as an initial investigation [5]. Supportive findings include a neutrophil leucocytosis, elevated transaminases and anemia. Diagnosis can be confirmed serologically by demonstrating the circulating antibodies specific to *Entamoeba histolytica*. Although it cannot differentiate acute from previous infections in endemic areas it has a high negative predictive value [3]. PCR has higher sensitivity in the diagnosis of ALA and bacterial culture of the aspirate can help to exclude a pyogenic abscess [6]. ALA is treated with nitroimidazoles, mainly metronidazole, and it is recommended to follow treatment with a luminally active amoebicidal drug. Aspiration, percutaneous drainage, and open surgical drainage are the modalities of interventional therapies available in complicated ALA.

We report a case of a patient who was found to have a right iliac fossa mass which was diagnosed as a localized collection of pus from a ruptured liver abscess which has been walled off. To our knowledge this is the first case ever reported of this kind. The case proves further the wide variety of clinical presentations of amoebic liver abscess and highlights that a high degree of clinical suspicion or vigilance is warranted in the diagnosis of this condition to prevent its potentially fatal complications.

## Case presentation

A 63-year-old farmer from the Northern Province of Sri Lanka presented to Teaching Hospital Jaffna, with a 3 week history of anorexia and loss of 6 kg of weight. He also had a 4 day history of fever with chills and rigors, associated with discomfort of the right lower abdomen and passage of frequent loose stools without blood or mucous. He had no significant past medical history. He drinks two bottles of toddy (palm wine), a popular local alcohol beverage, per day and has a 30 pack year history of cigarette smoking.

On examination, he was febrile, his pulse rate was 100 beats per minute, with a blood pressure of 90/60 mmHg. Abdominal examination revealed a tender hepatomegaly with a liver span of 15 cm in the mid clavicular line and an irregular liver edge and a large firm mass in the right iliac fossa (RIF). There was no guarding or rigidity noted. Bowel sounds were heard.

**Fig. 1:**
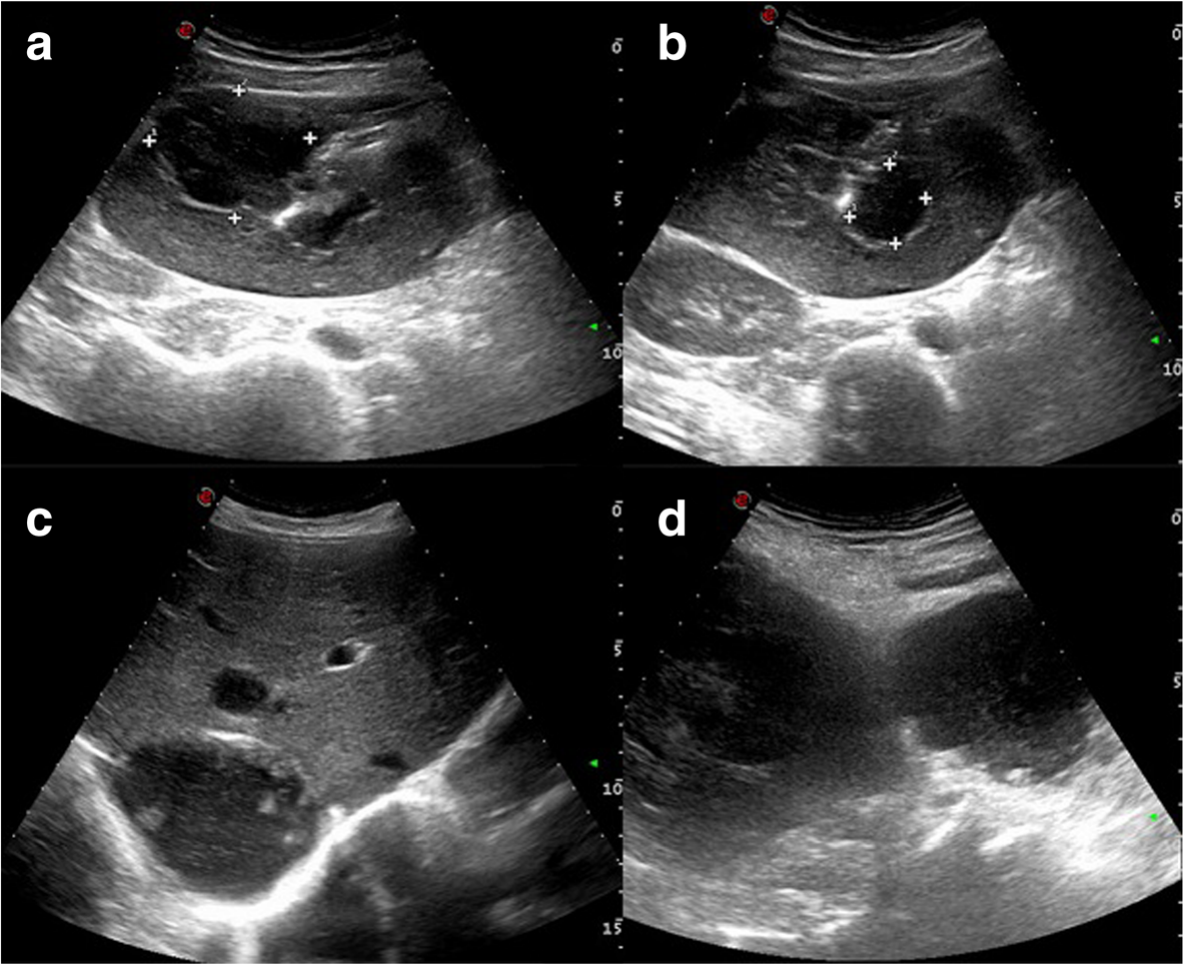
**a** right lobe liver abscess **b** left lobe abscess **c** localized collection in the right iliac fossa **d** liver abscess ruptured and leaking into the peritoneal cavity

After initial fluid resuscitation, an ultrasound scan of the abdomen was performed, which revealed two large liver abscesses - one in the right lobe measuring 7.0 × 7.2 cm and the other in the left lobe measuring 9.0 × 8.1 cm, leaking into the peritoneal cavity. A localized fluid collection was seen in the RIF measuring 5.0 × 5.9 cm (Fig. 1). Chest radiograph was normal with no evidence of pleural effusion or pneumonic patches.

Initial full blood count showed a neutrophil leucocytosis (White blood cell count – 14,000, neutrophils 77%, lymphocytes 15%, eosinophils 0.4%) and normocytic anemia with a hemoglobin of 9.4 g/dl and platelet count of 507,000. Liver profile was as follows: AST: 40 IU/L, ALT: 14 IU/L, Total bilirubin: 21.6 μmol/l, Direct bilirubin: 13.5 μmol/l. Prothrombin time was normal. Serum amylase was 47 U/L. ESR was 108 mm and the C-reactive protein was 192 mg/l. His random blood sugar levels were normal. Microscopic stool examination with wet smear and trichrome staining showed no evidence of trophozoite or cyst of *Entamoeba histolytica*. Circulating IgG antibody against *Entamoeba histolytica* was detected using the commercially purchased AccuDiagTM *E. histolytica* IgG (Amoebiasis) ELISA kit (USA).

**Fig. 2:**
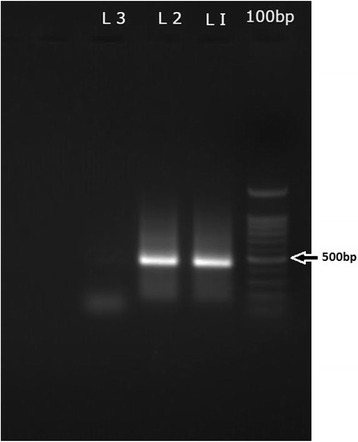
Results of nested PCR, amplified serine-rich protein coding gene (450 bps) of *Entamoeba histolytica*. L1: Positive control, L2: Left lobe Liver Abscess pus sample, L3: Negative control

He was treated with intravenous metronidazole. Ultrasound scan (USS) guided aspiration of both abscesses was done and 250 cc of anchovy sauce like pus was drained altogether. The pus culture was sterile for bacteria. For the PCR diagnosis, DNA was extracted from the pus sample obtained from the left lobe abscess, using the commercially available QIAGEN stool mini kit (Germany) as per the manufacturer’s instructions. Briefly, the initial PCR was performed using the extracted genomic DNA with the first set of primers SREHP-5 and SREHP-3 followed by the nested PCR using the 1:50 dilution of the initial PCR product as the template and the second set of primers nSREHP-5 and nSREHP-3 [6]. Gel electrophoresis analysis of the amplified PCR product with the positive control molecular biologically confirmed the presence of *Entamoeba histolytica* DNA in the abscess (Fig. 2). Shrinkage of the RIF fluid collection was noted ultrasonically immediately after the aspiration of pus from the left lobe abscess and previously seen firm mass in the right iliac fossa became less prominent on examination.

His loose stools and right lower abdominal discomfort improved on the next day of aspiration. Fever defervescence was noted on the third day of admission and he was discharged on the 5^th^ day with a course of oral metronidazole for a total duration of 14 days. He was given iodoquinol 650 mg eight hourly for 20 days to eradicate the intestinal colonization of the parasites. He made an uneventful recovery with complete resolution of the symptoms and the repeat ultrasound scan of the abdomen which was done after 2 months from admission confirmed complete resolution of both abscesses and RIF collection.

## Discussion

The diagnosis of ALA has undergone major changes with the application of imaging and molecular biology techniques. The new diagnostic techniques also lead to the identification of wide variety of clinical presentations and complications of ALA.

An amoebic liver abscess usually presents with abdominal pain, fever and anorexia [3].

Formation of an inflammatory mass or ‘amoeboma’ is a recognized but rare complication of intestinal amoebiasis, most commonly affecting the caecum and presenting as a mass in the RIF [7].

Paracaecal amoeboma presenting as a RIF mass has been reported previously [7]. The unusual presentation in this case was the finding of a RIF mass due to a ruptured liver abscess with a localized collection of pus which had been walled off. The absence of symptoms of amoebic colitis, negative stool microscopy for amoebic trophozoites and cyst, ultrasound evidence of liver abscess tracking into RIF fossa and immediate resolution of the RIF mass both clinically and radiologically, following aspiration of left lobe liver abscess supported that the RIF mass is not due to amoeboma, but due to the pus collection from the ruptured liver abscess.

RIF mass is a common diagnostic dilemma in clinical practice. Patients with RIF mass usually present with RIF pain, fever, vomiting, loss of appetite and loss of weight. According to a study conducted in South India among 50 patients with RIF mass, the most common cause was found to be appendicular lumps (mass/abscess) followed by ileocaecal tuberculosis and cancer of the caecum [8]. Other differential diagnoses to be considered are crohns disease, amoeboma, psoas abscess, actinomycosis and lymphoma.

Most patients with ALA don’t have coexistent intestinal amoebiasis at the time of presentation [9]. Although the microscopic stool examination for amoebic trophozoites or cysts, is a cost effective investigation in developing countries, the chance of detecting the parasite is very remote [10] Further the sensitivity of the microscopic examination of the aspirated pus sample in diagnosing ALA is also less than 20% [11].

ELISA is approved to be the standard serological reference test for the diagnosis of amoebiasis by the centre for disease control and prevention. Serological tests for anti amoebic antibodies are positive in nearly 90% of patients with ALA [12]. However the use of serology for diagnosis of amoebiasis in endemic population is of limited value due to the high prevalence of anti amoebic antibodies and inability to differentiate acute from previous infection [13]. Detection of the parasite by antigen detection or PCR in combination with serological tests or alone, though expensive, enhances diagnostic accuracy.

Our patient presented with a RIF mass, and ultrasound scan of the abdomen enabled the timely diagnosis of a ruptured liver abscess with localized pus collection. Although it is difficult to differentiate amoebic and pyogenic liver abscess ultrasonically, the sterile pus culture, positive amoebic serology and detection of *Entamoeba histolytica* DNA with PCR confirmed the diagnosis of ALA.

The prompt treatment with USS guided aspiration and administration of amoebicidal agents, prevented the potential rupture and peritonitis which could have lethal consequences.

## Conclusions

Recognition of the wide array of clinical presentations of amoebic liver abscess is crucial, especially considering the curable nature of this disease and the potentially devastating outcome of an untreated abscess. An intra-abdominal mass in a patient with an amoebic liver abscess, even when the mass is not in close vicinity to the liver, should raise the suspicion of a localized collection of pus and impending generalized peritonitis. Clinicians should be vigilant enough to think about the various rare presentations of extra intestinal amoebiasis in the endemic tropics.

Early utilization of imaging modalities coupled with application of battery of confirmatory serological and molecular studies whenever available can eliminate diagnostic dilemma in atypical presentations.

Early diagnosis and prompt intervention can prevent the dreaded complication of peritonitis and toxemia; hence reduce the consequent morbidity and mortality.

## Abbreviations

ALA: Amoebic liver abscess
ALT: Alanine transaminase
AST: Aspartate transaminase
DNA: Deoxyribonucleic acid
ELISA: Enzyme-linked immunosorbent assay
IgG: Immunoglobulin G
PCR: Polymerase chain reaction
RIF: Right iliac fossa
USS: Ultra sound scan

## References

[1] Hughe MA, Petri WA (2000). Amoebic liver abscess. Infect Dis Clin North Am.

[2] WHO (1997). WHO/PAHO/UNESCO. Consultation of experts on amoebiasis. Wkly Epidemiol Rec.

[3] Sharma MP, Ahuja V (2003). Amoebic liver abscess. JIACM.

[4] Terry Wuerz T, Kane JB (2012). A review of amoebic liver abscess for clinicians in a nonendemic setting. Can J Gastroenterol.

[5] Kimura K, Stoopen M, Reeder MM, Moncada R (1997). Amebiasis: modern diagnostic imaging with pathological and clinical correlation. Semin Roent.

[6] Ayeh Kumi PF, Ali IM, Lockhart LA, Gilchrist CA, Petri WA, Haque R (2001). Entamoeba histolytica: genetic diversity of clinical isolates from Bangladesh as demonstrated by polymorphisms in the serine-rich gene. Exp Parasitol.

[7] Davidson BR, Neoptolemos JP, Watkin D, Talbot IC (1988). Invasive amoebiasis: an unusual presentation. Gut.

[8] Shetty SK, Shankar M (2013). A clinical study of right iliac fossa mass. The internet journal of surgery.

[9] Juniper K, Worrel CL, Minshew MC, Roth LS, Cypert H, Lloyd RE (1972). Serological diagnosis of amoebiasis. Am J Trop Med Hyg..

[10] Chaudhary S, Noor MT, Jain S, Kumar K, Thakur BS. Amoebic liver abscess: a report from central India. Tropical Doctor. 2015 DOI 10.1177/0049475515592283. 10.1177/0049475515592283 26156972

[11] Haque R, Huston CD, Hughes M, Houpt E, Petri WA (2003). Amoebiasis. N Engl J Med.

[12] Espinosa-Cantellano M, Martinez-Palomo A (2000). Pathogenesis of intestinal amoebiasis: from molecules to disease. Clin Microbiol Rev.

[13] Martínez-Palomo A, Espinosa Cantellano M, Cox FEG, Kreier JP, Wakelin D (1998). Intestinal amoebae. Topley & Wilson's microbiology and microbial infections.

